# Influence of Semen Collection Frequency and Seasonal Variations on Fresh and Frozen Semen Quality in Thai Native Roosters

**DOI:** 10.3390/ani13040573

**Published:** 2023-02-06

**Authors:** Maruay Pimprasert, Theerapat Kheawkanha, Wuttigrai Boonkum, Vibuntita Chankitisakul

**Affiliations:** 1 Department of Animal Science, Faculty of Agriculture, Khon Kaen University, Khon Kaen 40002, Thailand; 2 Network Center for Animal Breeding and Omics Research, Faculty of Agriculture, Khon Kaen University, Khon Kaen 40002, Thailand

**Keywords:** season, semen collection frequency, rooster semen, Thai native chicken

## Abstract

**Simple Summary:**

Semen collection frequency and season are the primary factors influencing the ability to effectively utilize artificial insemination technology during rooster fertility to ensure semen quality and a usable number of sperm for cryopreservation. In this present study, we first conducted an experiment for one year to study the influence of semen collection frequency (once, twice, and thrice per week) and seasonal factors on the quality of fresh and frozen semen from Thai native roosters raised under an open-housed system. The results showed that neither fresh nor frozen semen quality was affected by semen collection frequency, but they were influenced by season. Semen collection can be conducted three times per week in native Thai roosters without affecting semen quality while maximizing sperm production. However, the rainy season negatively affected sperm concentration. The highest sperm production was obtained during the winter compared to the summer and the rainy season. Moreover, the decreased sperm motility of frozen-thawed semen obtained in the summer season might be related to the higher malondialdehyde concentration.

**Abstract:**

This study aimed to determine the effects of the frequency of semen collection (once, twice, and thrice weekly) and seasonal variations on the fresh and frozen semen quality of Thai native roosters throughout the year. Data on temperature and humidity were collected and used to calculate the temperature–humidity index (THI). The average temperature and THI were lower in the winter than in the rainy season and the summer (*p* < 0.05). In contrast, the average relative humidity was not different among the seasons but was higher in the rainy season (*p* > 0.05). None of the fresh or frozen semen quality parameters were influenced by the frequency of semen collection, but the season did have an effect. The highest sperm concentration was obtained in the winter (*p* < 0.05). In contrast, the lowest sperm concentration was found during the rainy season, which presented the highest humidity. Regarding the frozen semen quality, the highest malondialdehyde concentration and the lowest motility were found in the summer (*p* < 0.05). In conclusion, semen collection can be conducted thrice per week for a consecutive year without affecting semen quality while maximizing sperm production. However, the highest sperm production was obtained in the winter, which is also a suitable season for producing semen for cryopreservation.

## 1. Introduction

Thai native chickens, especially Pradu Hang dum (PD), are very popular with consumers because of the unique texture of their meat [1,2] and the potential substances it contains [3,4]. Their production system is currently evolving from backyards to battery cages for parent stocks because of several advantages the latter provides, such as a high feed density, ease of management, high egg production, and an increased mating ratio [5]. Additionally, the artificial insemination (AI) procedure has become more familiar to Thai farmers. However, a study showed that rooster fertility on the farm under field conditions was lower than that under experimental conditions [6]. One important concern is that local farmers have no practical method for mating management. To effectively utilize the full potential of AI technology during rooster fertility, semen collection frequency [7] and season, considered primary factors influencing the productivity and profitability of hatcheries, are taken into consideration by farmers and are easy for them address at the farm.

Chicken sperm are produced in the intra-abdominal testes at a core body temperature (40–41 °C) for 14 days and then are transported through the epididymis to the vas deferens, where they are stored for 2–3 days and undergo some natural degeneration before being exported at ejaculation [8]. In light of this physiological evidence, it is reasonable to collect semen at a consistent frequency to maintain optimal sperm quality during fertility. Twice-a-week collection theoretically results in maximal output for broiler breeders but not for all roosters [9]. Six collections per week in Taiwanese chickens enhanced the total sperm and motile sperm production in a week [10]. In contrast, more than three rounds of collections per week in turkeys decreased the semen volume [11]. Native Thai farmers generally collect semen once or twice per week as a regular practice, which is too low compared to previous studies.

In addition to the frequency of semen collection, adverse climate conditions severely affect semen production, as semen volume and sperm concentration have been shown to be reduced during hot climatic conditions in other poultry. The lowest semen volume and concentration values of Shikabrown breeder cocks are 0.39 mL and 2.90 × 10^9^ sperm per mL in the hot–dry season [12]. In broiler roosters, a significantly reduced sperm concentration (3.21 × 10^9^ sperm per mL) occurs in the first week of heat stress [13]. A commercial rooster (White Leghorn) raised in India exhibited superior reproductive performance during the winter [14]. However, a study on seasonal variation in semen quality has never been reported in Thai native chickens.

Therefore, the objective of this study was to identify the ideal frequency of semen collection, i.e., the one with no adverse effects on semen quality, and the duration of collection over a year in Thai native chickens. For this purpose, the present study evaluated the influence of semen collection frequencies (once, twice, and thrice per week) on semen characteristics such as volume, sperm motility, sperm viability, and sperm concentration during a year of semen collection during three seasons (summer, rainy, and winter).

Semen cryopreservation is a preferable procedure for the ex situ management of the genetic diversity of purebred animals [15]. However, the reported success rates are variable. The problem is mainly due to the chicken sperm plasma membrane containing high amounts of polyunsaturated fatty acids (PUFAs), which tend to make sperm cells susceptible to harmful reactive oxygen species (ROS) [16]. In addition, many studies have revealed that high ambient temperature causes oxidative stress by producing ROS [17]. Therefore, in this present study, after fresh semen evaluation, semen samples were cryopreserved to determine the effects of semen collection frequency and season on Thai rooster semen cryopreservation. The malondialdehyde (MDA) concentration, commonly known as a marker of oxidative stress that occurs from lipid peroxidation following semen cryopreservation processing, was measured in the semen samples.

## 2. Materials and Methods

### 2.1. Animals

A total of 36 native Thai roosters (Pradu Hang dum) at 27 weeks of age were reared at the experimental unit of the Network Center for Animal Breeding and Omics Research, Khon Kaen University, Thailand. The roosters were housed in cages measuring 45 × 50 × 60 cm in an open-housed system exposed to natural daylight and temperature. They were fed 100 g/day of commercial diet, and freshwater was available ad libitum throughout the experimental period. All animal procedures were approved by the Institutional Animal Care and Use Committee based on the Ethics of Animal Experimentation of the National Research Council of Thailand (record no. IACUC-KKU-130/64; reference no. 660201.2.11/653, and record no. IACUC-KKU-131/64; reference no. 660201.2.11/654).

### 2.2. Experimental Design

The roosters were randomly divided into three treatment groups (12 roosters per group) according to the frequency of semen collection. Treatment 1 (T1): once weekly (Monday); Treatment 2 (T2): twice weekly (Monday and Wednesday); and Treatment 3 (T3): thrice weekly (Monday, Wednesday, and Friday). The semen samples were collected for 52 weeks at between 27 and 71 weeks of age (total of 12 months). The semen characteristics were examined and recorded in terms of semen volume, mass movement, sperm concentration, and sperm viability. Then, the semen samples were pooled for cryopreservation. The frozen-thawed semen was assessed in terms of total motility (MOT), progressive motility (PMOT), sperm viability, and MDA concentration.

The seasonal determination followed the report from the Meteorological Department of Northeast Thailand (2020–2021). Based on its recommendation, the months of March to May were classified as the summer season, June to October as the rainy season, and November to February as the winter season. Data on average daily temperature and relative humidity were recorded using an automatic temperature and humidity meter (data logger; EL-USB-2, Lascar Electronics, Whiteparish, England) at the farm area to calculate the temperature–humidity index (THI), which is associated with the level of thermal/heat stress. The THI formula [18] used is shown below, with temp as the ambient temperature (°C) and RH as the relative humidity (%).

THI = (1.8 × temp + 32) − (0.55 − 0.0055 × RH) × (1.8 × temp − 26)

### 2.3. Semen Collection

The semen samples were collected into a 1.5 mL Eppendorf tube containing 0.1 mL of Schramm diluent [19] using dorsal-abdominal massage. The semen samples were protected from light and kept at a temperature of 22–25 °C during transport to the laboratory within 20 min after collection for macroscopic and microscopic evaluation. Semen collection was always performed by the same person to maximize semen quality and quantity, and was handled carefully to prevent cross-contamination during semen collection.

### 2.4. Fresh Semen Evaluation

The semen volume was measured by drawing the sample into a tuberculin syringe (Nipro (Thailand) Co., Ltd., Phra Nakhon Si Ayutthaya, Thailand. The intensity of the waves formed by sperm movements was assessed. Immediately after semen collection, a drop of semen sample was placed on a slide without a coverslip, examined under a compound microscope (100×) and scored on a scale of 1–5 (0 = no sperm movement; 5 = very rapid waves and whirlpools visible, with more than 90% of sperm showing a forward movement).

The sperm concentration was determined using a hemocytometer counting chamber (Lo-Laboroptik Ltd., Lancing, West Sussex, England). Five microliters of semen sample was diluted with 195 µL of sodium chloride. A drop of semen sample was then placed on a hemocytometer, and the reading was recorded under a compound microscope (400× magnification). The sperm concentration was expressed as billion (10^9^) sperm cells/mL.

The total sperm number per ejaculate was calculated as the semen volume multiplied by the sperm concentration. The weekly total semen volume was calculated as the semen volume multiplied by the time to semen collection. Weekly sperm production was calculated as the ejaculated volume production and ejaculated frequency.

### 2.5. Semen Cryopreservation and Thawing

After fresh semen evaluation, semen that passed the criteria 0.2–0.6 mL of volume, sperm concentration ≥3 × 10^9^ sperm/mL, and motility score ≥ 4 were pooled to eliminate individual differences for further cryopreservation processing.

The pooled semen was diluted 1:2 in a semen extender at 25 °C and then cooled from 25 to 5 °C for 1 h. Afterward, DMF (N,N-dimethylformamide) (Sigma-Aldrich, Inc., St. Louis, MO, USA) was added to the semen extender (at a final concentration of 6% DMF) in the diluted semen samples. Then, the semen was immediately loaded into 0.5 mL plastic straws and equilibrated at 5 °C for 15 min. Before immersion into liquid nitrogen (LN_2_), the semen straws were placed at 11 cm and 3 cm heights above the surface of LN_2_ in vapor for 12 min and 5 min, respectively. Semen straws were stored in an LN_2_ container until thawing. For thawing, the straws were placed in cool water at 5 °C for 5 min.

### 2.6. Postthaw Semen Evaluation

A computer-assisted semen analysis (CASA) system (Hamilton Thorne Biosciences, version 10 HIM-IVOS, Beverly, MA, USA) was used to evaluate sperm motility, i.e., total motility (MOT) and progressive motility (PMOT). The CASA was set up as described in our previous study [20] using the following settings: frames per second, 60 Hz; minimum contrast, 25; minimum cell size, 4 pixels. Semen samples were diluted in Schramm at a ratio of 1:15, and 6 µL of semen was loaded into a 2× cell slide. At least 300 sperm were evaluated. The percentages of MOT and PMOT were recorded.

Dual fluorescent staining using SYBR-14 and propidium iodide (PI) kits (Live/dead sperm viability kit L7011; Invitrogen, Thermo Fisher Scientific, Waltham, MA, USA) was used to analyze sperm viability [21]. In brief, 150 µL semen was incubated at 5 °C for 10 min with 5 µL of SYBR-14. Then, 5 µL of PI was added and mixed well before incubation for 5 min. Viability was assessed under an IX71 fluorescence microscope (Olympus, Tokyo, Japan) at 400× magnification by counting at least 200 spermatozoa. A bright green sperm cell was classified as an intact plasma membrane, while sperm with a damaged plasma membrane appeared red. Sperm viability was expressed as the percentage of live sperm with intact plasma membranes.

The concentration of MDA is an index of lipid peroxidation in semen samples. It was measured using the thiobarbituric acid (TBA) reaction described in our previous study [21]. A 250 µL semen sample (250 × 10^6^ spz/mL) was incubated at 37 °C for 60 min with 0.25 mL of ferrous sulfate and 0.25 mL of sodium ascorbate. Then, 1 mL of trichloroacetic acid (15% (*w*/*v*)) and 1 mL of TBA (0.375% (*w*/*v*)) were added and boiled for 10 min. The samples were subsequently cooled at 4 °C to stop the reaction. Finally, the samples were centrifuged at 4 °C and 5000× *g* for 10 min to separate the sperm pellets and the supernatant. The absorbance in the supernatant (2 mL) was determined at 532 nm using a spectrophotometer (UV-1200, Shimadzu, Japan).

### 2.7. Statistical Analysis

Data were analyzed using SAS v. 9.0 statistical software (SAS Institute, Inc., Cary, NC, USA) [22]. First, data were tested for normality and homogeneity of variance and then analyzed using PROC ANOVA with a completely randomized design (CRD) using repeated measures. The study factors were semen collection frequency and season and their interaction. Each factor was compared for differences using Tukey’s post hoc test. Differences were considered significant when *p* < 0.05.

## 3. Results

The monthly data on temperature, relative humidity, and THI of each season are presented in Table 1. The average temperature was significantly lower in the winter (25.63 °C) than in the rainy season (29.28 °C) and the summer (29.80 °C) (*p* < 0.05). The average relative humidity was not significantly different among the seasons but was higher in the rainy season (73.09%; *p* > 0.05). The average THI was the lowest in the winter (74.68; *p* < 0.05), while the THI during the summer and rainy seasons did not differ (81.06 and 80.54, respectively).

### 3.1. Fresh Semen Quality

The levels of significance of the influence of semen collection frequency and season on fresh semen quality are shown in Table 2. The interaction effect between semen collection frequency and season was nonsignificant for any semen quality parameter. Only the effect of season was significant for the sperm concentration and average sperm number (*p* < 0.01), while semen collection frequency did not influence any parameter (*p* > 0.05).

**Figure 1:**
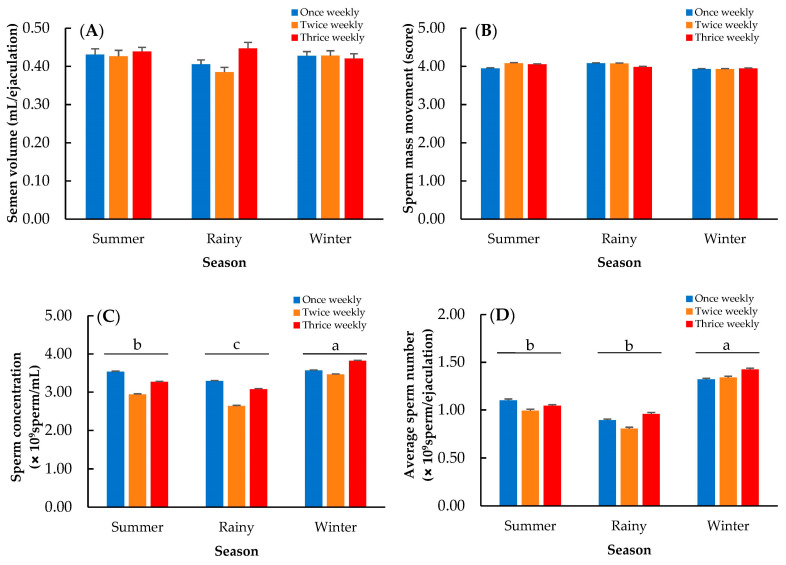
Effect of season on semen volume (**A**), sperm mass movement (**B**), sperm concentration (**C**), and average sperm number (**D**); a, b, c: means followed by different letters differ significantly among seasons at *p* < 0.01.

Data summarizing the effect of season on semen quality at each semen collection frequency are shown in Figure 1. The semen volume and sperm movement did not differ among seasons (Figure 1A,B; *p* > 0.05). The sperm concentration was significantly lower on rainy days. Moreover, the highest sperm concentration was obtained in the winter (Figure 1C; *p* < 0.01). The average sperm production followed the pattern observed for sperm concentration and is shown in Figure 1D.

**Figure 2:**
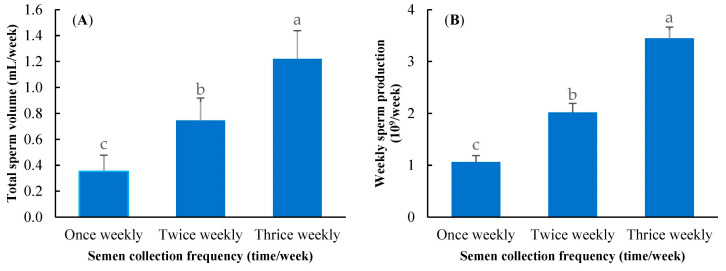
Effect of semen collection frequency on total sperm volume (**A**) and weekly sperm production (**B**); a, b, c: means followed by different letters differ significantly among semen collection frequencies at *p* < 0.01.

The highest total semen volume and weekly sperm production were obtained in the thrice-weekly sperm collection (*p* ≤ 0.01), while these parameters were lowest in the once-weekly sperm collection (*p* ≤ 0.01), as shown in Figure 2.

### 3.2. Frozen Semen Quality

**Figure 3:**
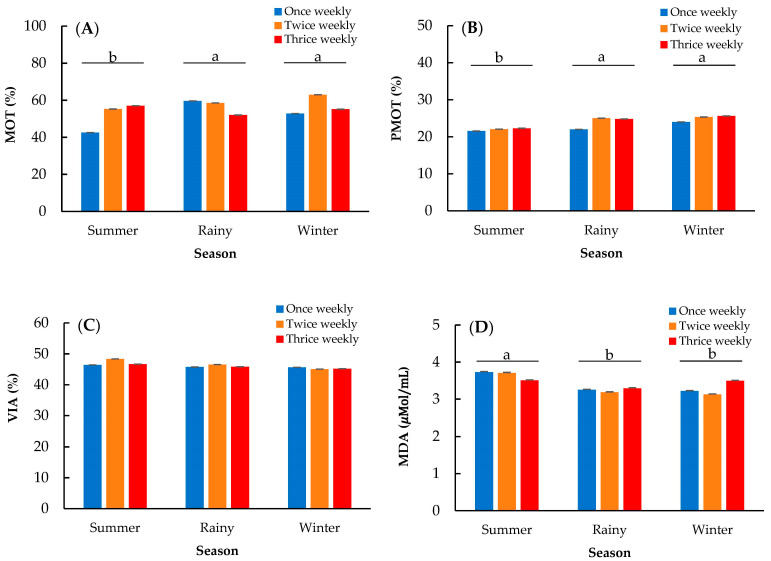
Effect of season on total motility (MOT; (**A**)), progressive motility (PMOT; (**B**)) sperm viability (VIA; (**C**)), and malondialdehyde (MDA; (**D**)) concentration in frozen-thawed rooster semen. a, b means followed by different letters differ significantly among seasons at *p* < 0.05.

Table 3 shows the mean parameters of frozen-thawed sperm quality. The interaction effect between semen collection frequency and season was nonsignificant for all semen quality parameters. There were no differences among the frequencies or seasons for sperm viability (Figure 3C; *p* > 0.05). However, MDA concentration and sperm motility were affected by the season. The highest MDA concentration was found in the summer (3.64 ± 0.07 µMOL/mL; *p* < 0.01), while those in the rainy and winter seasons were 3.24 ± 0.05 µMOL/mL and 3.28 ± 0.06 µMOL/mL, respectively (Figure 3D). The total motility and progressive motility followed the pattern observed for MDA concentration, in which the lowest sperm motility was found in the summer (*p* < 0.05), while those in the rainy and winter seasons were not different (Figure 3A,B).

## 4. Discussion

It is important to establish an ideal semen collection frequency to ensure the presence of high-quality semen, which is required for greater AI efficiency. Twice-a-week collection is theoretically the maximal output in broiler breeders [9]. Six collections per week in Taiwan chickens enhances the weekly total sperm and motile sperm production [10]. In contrast, more than three rounds of collections per week in turkeys decreased the semen volume [11]. However, those studies were conducted for a short time (4–13 experimental weeks). This study revealed that semen collection could be conducted three times per week throughout the year in native Thai roosters without affecting semen quality (Table 2). In addition, the increase in semen collection frequency increases the semen yield per week (Figure 2), meaning that the number of doses available for insemination will be greater than at one/two rounds of collection per week. This information is advantageous for further determining the optimal male-to-female ratio in breeder flocks under field conditions.

The THI represents the combined effects of environmental temperature (°C) and relative humidity (%) and is widely used to measure the impact of heat stress. THI values exceeding a threshold of 76 seem to decrease the growth performance of Thai native chickens [23]. However, information on heat stress and reproductive status is limited; to date, there are no studies that determined the THI levels affecting semen quality in chickens. Therefore, in this present study, we recorded the ambient temperature and humidity for THI calculation in each season, which was used as one of the factors that could affect semen quality in the experiment. The sperm concentration was significantly greater during the winter (THI 74.68) than during the summer (THI 81.06) and rainy (THI 80.54) seasons (Figure 1). Exposure to heat stress at a temperature greater than 32 ± 1 °C with a humidity of 55–65% could induce spermatogenic cell abnormalities in chicken testes and decrease testosterone production [24,25]. Testosterone is necessary for spermatogenesis [26]; a higher level of testosterone in chickens was observed during the winter, and this might be responsible for a higher sperm concentration [14]. Wannaratana et al. [27] and Karaca et al. [28] also reported similar findings on increased sperm production during the winter in pigeons, turkeys, and White Leghorns. Therefore, it is inferred that a low ambient temperature in the winter (at THI 71–76) leads to an increase in sperm production. Moreover, the lowest sperm concentration was found not during the summer (at THI 79–82) but during the rainy season (at THI 78–81), which had the highest humidity level (73.09%) (Table 1), suggesting the adverse effect of humidity, as reported in pigeons by Wannaratana et al. [27], who showed that sperm concentration was also negatively affected by high humidity (>72.5%). Although studies on the effects of humidity on the reproductive performance and physiology of roosters are limited, the negative effect of high humidity may be explained by the fact that it reduces respiratory efficiency and moisture evaporation [29]. Heat stress can result in panting to promote heat loss; however, a high humidity level decreases moisture evaporation by panting [30], making roosters more prone to heat stress. Subsequently, these roosters are highly affected and decrease their semen production.

High amounts of PUFAs in the sperm plasma membrane result in susceptibility to the harmful effects of ROS during cryopreservation, which reduces sperm membrane quality and fertility potential [31]. In addition, many studies have revealed that high ambient temperature causes oxidative stress by producing ROS [17]. In the current study, the semen from each collection frequency was cryopreserved under similar conditions; therefore, the quality of frozen-thawed semen in each semen collection frequency should not be different. Thus, we hypothesized that the lipid peroxidation following semen cryopreservation processing would be different among seasons by being greater in the summer, which had the highest ambient temperature and THI. Thus, the MDA concentration of frozen-thawed semen was assessed and compared among seasons. The results revealed that MDA was significantly higher in the summer season, resulting in decreased sperm motility (Figure 3). The MDA level is commonly known as a marker of oxidative stress, which has been evaluated in many studies to indicate lipid peroxidation in the samples. The natural antioxidants that contribute to seminal plasma are responsible for protecting the sperm plasma membrane from oxidative damage by scavenging excess ROS during cryopreservation processing [32]. However, it was found that the levels of natural antioxidants decreased after semen collection, and rooster sperm were exposed to chilling injuries, leading to membrane damage throughout cryopreservation. This may result from an imbalance between ROS generation and inadequate scavenging ability of the cellular antioxidant system during in vitro preservation [33]. In addition, the seminal plasma from heat-stressed roosters was shown to contain lower ionic Ca, Na+, and Cl-; therefore, ions were fluxed between sperm and seminal plasma, decreasing intracellular sperm Ca concentration [28]. Intracellular sperm Ca plays a significant role in sperm motility and fertility [34,35]. Lower antioxidant enzyme activity together with higher lipid peroxidation in seminal plasma was observed in the summer than in the winter [14]. In other words, heat stress caused a decrease in antioxidant enzyme activity and disrupted the balance between the oxidative and antioxidative status [25]. Thus, we infer that a higher environmental temperature during the summer accelerates lipid peroxidation during cryopreservation processing, resulting in an increase in ROS and, subsequently, a decline in sperm motility. Therefore, antioxidant supplementation of diets [20] or use of freezing extenders [36,37] is recommended to reduce lipid peroxidation and improve semen cryopreservation under high environmental temperatures.

## 5. Conclusions

We report for the first time in this study that semen collection frequency does not affect fresh or frozen semen quality in native Thai roosters. Thus, semen collection can be conducted three times per week for a consecutive year without affecting semen quality while maximizing sperm production. This information is advantageous for further determining the optimal male-to-female ratio in breeder flocks under field conditions. Additionally, we found that the rainy season negatively affected sperm concentration. Moreover, the highest sperm production rate was found in the winter, which is a season suitable for semen cryopreservation.

## Figures and Tables

**Table 1:** Monthly data on each season’s temperature, relative humidity, and THI during the experimental period.

Season	Month	Temperature (°C)	Relative Humidity (%)	THI
Summer	March	28.71	69.90	79.39
	April	30.04	69.42	81.26
	May	30.66	71.41	82.52
Rainy	June	29.13	74.07	80.48
	July	30.11	70.56	81.53
	August	29.40	73.82	80.70
	September	30.11	70.56	81.53
	October	27.67	76.42	78.45
Winter	November	26.73	70.14	76.23
	December	23.68	64.71	71.03
	January	24.94	66.92	73.02
	February	25.12	64.61	73.41
Mean				
Summer		29.80 ^a^	70.24	81.06 ^a^
Rainy		29.28 ^a^	73.09	80.54 ^a^
Winter		25.63 ^b^	68.56	74.68 ^b^

^a,b^ Different letters within a column indicate significant differences (*p* < 0.05).

**Table 2:** Effect of semen collection frequency and season and their interaction on semen volume, mass movement, sperm concentration, and average sperm number of fresh rooster semen.

Parameter	Weekly Frequency			Mean ± SE	Semen Collection Frequency (a)	Season (b)	Interaction a∗b
	1	2	3				
Semen volume (mL/ejaculation)	0.42 ± 0.01	0.41 ± 0.02	0.44 ± 0.01	0.42 ± 0.01	0.151	0.307	0.192
Mass movement (score)	3.98 ± 0.06	4.04 ± 0.06	4.00 ± 0.05	4.00 ± 0.06	0.621	0.101	0.618
Sperm concentration (×10^9^ sperm/mL)	3.48 ± 0.12	3.18 ± 0.13	3.39 ± 0.11	3.29 ± 0.12	0.081	0.001	0.416
ASN (×10^9^ sperm/ejaculation)	1.10 ± 0.09	1.03 ± 0.09	1.13 ± 0.09	1.10 ± 0.09	0.448	0.001	0.944

ASN = average sperm number; *p* < 0.05.

**Table 3:** Effect of semen collection frequency and season on total motility (MOT), progressive motility (PMOT), sperm viability (VIA), and malondialdehyde (MDA) concentration of frozen rooster semen.

Parameter	Frequency			Mean ± SE	Semen Collection Frequency (a)	Season (b)	Interaction a∗b
	1	2	3				
MOT (%)	51.63 ± 1.79	56.90 ± 1.84	54.72 ± 1.80	55.08 ± 1.74	0.583	0.023	0.325
PMOT (%)	23.00 ± 1.21	26.79 ± 1.27	25.88 ± 0.97	25.22 ± 1.15	0.103	0.016	0.282
VIA (%)	45.24 ± 0.88	45.96 ± 1.10	45.20 ± 1.00	45.47 ± 0.99	0.805	0.574	0.804
MDA (µMol/mL)	3.40 ± 0.64	3.34 ± 0.09	3.43 ± 0.12	3.39 ± 0.28	0.606	0.001	0.257

## Data Availability

Not applicable.
